# Transition to retirement impact on risk of depression and suicidality: results from a longitudinal analysis of the Survey of Health, Ageing and Retirement in Europe (SHARE)

**DOI:** 10.1017/S2045796023000239

**Published:** 2023-05-11

**Authors:** G. Mosconi, G. P. Vigezzi, P. Bertuccio, A. Amerio, A. Odone

**Affiliations:** 1 Department of Public Health, Experimental and Forensic Medicine, Università degli Studi di Pavia, Pavia, Italy; 2 Ca’ della Paglia College, Fondazione Ghislieri, Pavia, Italy; 3 Department of Neuroscience, Rehabilitation, Ophthalmology, Genetics, Maternal and Child Health, Università degli Studi di Genova, Genoa, Italy

**Keywords:** depression, elderly, risk factors, social and political issues, suicide

## Abstract

**Aims:**

Depression is among the main contributors to older adults’ mental health burden. Retirement, one of the major life transitions, has been claimed to influence mental health substantially. Following up on a previous meta-analysis, the study aims to assess from a longitudinal perspective short- and long-term impacts of transitioning to retirement on depression risk and suicidality in older adults across Europe.

**Methods:**

We conducted a longitudinal study using data from the Survey of Health, Ageing and Retirement in Europe (SHARE), collected between 2004 and 2020 in 27 European countries plus Israel. To estimate relative risks (RR) and 95% confidence intervals (95% CIs) for depression and suicidality at seven time intervals before and after retirement, we fitted adjusted generalized estimating equation models for repeated measures.

**Results:**

We included 8,998 individuals employed at baseline and retired at follow-up (median follow-up time: 9 years; maximum: 16 years). Compared to the year of retirement, the risk of depression was 11% lower in the following year (RR 0.89; 95% CI 0.81–0.99), 9% lower after 2 years (RR 0.91; 95% CI 0.82–1.00) and after 3 years (RR 0.91; 95% CI 0.81–1.01). Significant estimates remained among females, married individuals, those with an intermediate or higher level of education, former manual workers and those who retired at or before their country’s median retirement age. A significant increase in depressive symptoms emerged from the tenth year after retirement among former non-manual workers (RR 1.21; 95% CI 1.05–1.40) and late retirees (RR 1.37; 95% CI 1.16–1.63). No heterogeneity emerged among strata. As for suicidality, we reported an increase in risk only 5 years or more after retirement, namely +30% 5–9 years after retirement (RR 1.30; 95% CI 1.04–1.64) and +47% 10 or more years after retirement (RR 1.47; 95% CI 1.09–1.98). Sensitivity analyses excluding subjects who reported a diagnosis of depression over the study period and those retirees who declared to receive a disability pension confirmed the results obtained in the overall analysis.

**Conclusions:**

Longitudinal adjusted data suggest an independent effect of retiring associated with a reduction in depression and suicidality risk in the short run, with its effect decreasing in the long run. Such trends are particularly evident among selected subgroups of elderly populations. If greater flexibility in pensionable age may help prevent depression late in life, the transition to retirement is to be accompanied by targeted health promotion interventions. In an ageing society, welfare policies should be evaluated, considering their long-term impact on mental health.

## Introduction

Low fertility and increasing longevity are causing the European population to grow older rapidly, with a constant rise in the share of retirees against that of working-age people (Eurostat, 2019). Despite the increase in lifespan, the proportion of life we spend in good health has not significantly changed in the last three decades, meaning we spend more years of our lives dealing with illness and disability than in the past (Vos *et al.*, 2020; WHO, 2021). The current demographic transition has profound implications for our society, raising questions about the sustainability of health and social welfare systems and posing new challenges in public policymaking, with particular reference to retirement (Eurostat, 2019).

In this scenario, identifying the determinants of healthy ageing is of crucial importance (Abud *et al.*, 2022; WHO, 2021). In older age, mental disorders represent one of the primary causes of illness and disability and are related to an increased risk of chronic physical diseases, suicide and all-cause death (Scott *et al.*, 2016; Vos *et al.*, 2020). Among them, depression is the leading contributor to the overall global burden of mental diseases (Vos *et al.*, 2020), affecting up to one in three adults over the age of 60 (Hu *et al.*, 2022; Zenebe *et al.*, 2021). Despite the fact that depression tends to decrease significantly among older adults (WHO, 2017), recent evidence reports a progressive increase in suicide rates with age (Alicandro *et al.*, 2019), especially among men (Shah *et al.*, 2016), suggesting only partially overlapping pathogenesis (De Leo *et al.*, 2013). Even though mental health disorders’ mechanisms in older life are not fully understood (De Leo, 2022), late-life well-being is recognised as the result of a complex interaction of biological, social, environmental and psychological factors with multiple life-course determinants having a role as risk factors or as protective factors (Mendonca Lima, 2011).

It has been claimed that retirement, one of life’s major transitions and related social determinants, has a significant impact not only on lifestyles but also on mental health (Henning *et al.*, 2016; Vigezzi *et al.*, 2021; Westerlund *et al.*, 2009). Indeed, retirement might involve either positive or detrimental changes in psychological well-being, including depressive symptoms and suicidal ideation (Bossé *et al.*, 1991; Page *et al.*, 2021). On the one hand, retiring is a potentially stress-generating event and might determine retirees’ loss of interpersonal relationships, consolidated routines and social position and role (Portnoi, 1981; Riley and Riley Jr, 1994; Zeduri *et al.*, 2022). On the other hand, the increase in free time to engage in social interactions and healthy activities adds to the relief from work-related risk factors (d’Errico *et al.*, 2022), especially for demanding jobs (Eibich, 2015; van der Heide *et al.*, 2013).

We recently conducted a systematic review and meta-analysis, pooling data from 41 original studies and suggesting a 20% protective effect of retirement on depressive symptoms. However, there was high heterogeneity between risk estimates; most studies had a cross-sectional design, which did not allow for exploring causality, and, on top of that, it was not possible to differentiate between the potential risk of depression for short- or long-term exposure to retirement (most of the included longitudinal studies had less than 10 years of follow-up) (Odone *et al.*, 2021). Concerning suicidality, evidence of association between an increased risk of suicide and retirement is limited and mainly relies on unrepresentative cross-sectional studies (Harwood *et al.*, 2006; Minayo *et al.*, 2012; Schneider *et al.*, 2011). In considering the complex and bidirectional relationship that links work, retirement and health (Oksanen and Virtanen, 2012) and the previously conceptualised frameworks on the topic (Kuhn, 2018; van Solinge, 2007), we aimed to fill such research gaps by analysing longitudinal data from a large cohort derived from the Survey of Health, Ageing and Retirement in Europe (SHARE) (Börsch-Supan *et al.*, 2013). In particular, our objective was to define and quantify the effect of retirement on depressive symptoms and suicidality, as well as to track the temporal evolution of these associations.

## Methods

### Study design and data source

Using individual-level SHARE data, we built a longitudinal cohort to investigate the impact of the transition to retirement on depressive symptoms and suicidality and their determinants, stratifying results according to individual and contextual characteristics. The SHARE project is based on an extensive and comprehensive data collection, including cross-sectional and longitudinal individual-level data on current characteristics, behaviours and retrospective life histories of people aged 50 years or older from 28 European countries plus Israel, collected in biennial waves since 2004. To enable cross-country comparisons, SHARE data are collected through homogeneous computer-assisted personal interviews using a questionnaire covering several domains, including information on health, socioeconomic status and family environment. Every aspect of the data generation process is carried out according to defined standards. Additionally, ex post harmonisation allows to overcome the problems associated with the international variability of country-specific variables and measurements (Börsch-Supan *et al.*, 2013). SHARE protocol, study design and all study-related details can be retrieved elsewhere (Börsch-Supan *et al.*, 2013, 2005).

### Data linkage

As done before (Bertuccio *et al.*, 2023), through a record-linkage procedure, we pooled individual-level data of SHARE waves 1 to 8, covering the period from 2004 to 2020. We combined three publicly available datasets for each wave, including data on sociodemographic traits (module DN), behavioural aspects (module BR) and job and pension variables (module EP). To gather harmonised data on education, occupation and health, we also integrated three additional databases, including the so-called ‘generated variables’ (i.e., the gv isced, gv isco and gv health databases). A key variable, named *mergeid*, allows a constant and unique identification of all participants through all the waves. By merging longitudinal micro-data from all the waves 1 to 8, we built a cohort of European subjects aged 50 years or older who were employed at baseline and transited to retirement at follow-up.

#### Variables of interest

The exposure of interest was time (in years) before and after retirement, computed as the difference between the year of retirement and the year of the interview. Time before and after retirement was split into seven periods: 10 years or more before retirement, from 9 to 5 years before retirement, from 4 to 1 year before retirement, the year of retirement (i.e., time 0), from 1 to 4 years after retirement, from 5 to 9 years after and 10 years or more after retirement. The year of retirement was considered as the reference category.

The primary outcome of interest was the risk of depression, as derived from the EURO-D scale (Maskileyson *et al.*, 2021; Prince *et al.*, 1999). The EURO-D is a score obtained by summing the presence in the 4 weeks before the interview of 12 symptoms, including depressed mood, pessimism, suicidality, guilt, sleep, interest, irritability, appetite, fatigue, concentration, enjoyment and tearfulness. The optimal cutoff point on the EURO-D scale for prediction of Geriatric Mental Scale depression (DN, neurotic clinical depression or DP, psychotic clinical depression) and SHORT-CARE (Comprehensive Assessment and Referral Evaluation) pervasive depression is 4, with high positive predictive values (Prince *et al.*, 1999). Therefore, we considered it a binary outcome with a score of 4 or more predicting the risk of depression (Prince *et al.*, 1999). In addition, we considered suicidality (yes vs. no) as the secondary outcome of our study, and single component of the EURO-D scale, which expresses suicidal feelings or the wish to be dead.

We considered the following variables as covariates: geographical area, sex, age at baseline, marital status (married/cohabiting, divorced/widowed and never married) at baseline, the highest level of education attained, occupational category at baseline and the current presence of at least one chronic disease (yes vs. no). Education was classified into three levels following the International Standard Classification of Education (ISCED): low (ISCED levels: 1–3), intermediate (ISCED levels: 5–6) and high (ISCED levels: 7–8). To define the occupational category, we considered the 10 primary groupings of the International Standard Classification of Occupations (ISCO; ILO, 2012): (1) managers, (2) professionals, (3) technicians and associate professionals, (4) clerical support workers, (5) services and sales workers, (6) skilled agricultural, forestry and fishery workers, (7) craft and related trades workers, (8) plant and machine operators and assemblers, (9) elementary occupations and (10) armed forces. European countries have been grouped according to the World Bank classification (TheWorld Bank, 2022) into the following geographical areas: North (including Denmark, Finland and Sweden), West (Austria, Belgium, France, Germany, Luxembourg, the Netherlands and Switzerland), South (Cyprus, Greece, Italy, Malta, Portugal and Spain), East (Bulgaria, Croatia, Czechia, Estonia, Hungary, Latvia, Lithuania, Poland, Romania, Slovakia and Slovenia), plus Israel.

Finally, we constructed a variable to classify the whole cohort into two groups based on the vulnerability of each individual to mental health disorders. The ‘vulnerable group’ was identified as the subgroup of individuals who declared ever to be treated for depression by a doctor or psychiatrist (wave 1, 2 and 4) or have had a diagnosis of affective or emotional disorders in the past or at the time of interview (wave 5, 6, 7 and 8).

#### Statistical analysis

To estimate the relative risk (RR) and the corresponding 95% confidence intervals (95% CI) of obtaining a EURO-D score ≥4 (i.e., primary outcome) and reporting suicidal thoughts (i.e., secondary outcome) at different time intervals (in years) before and after retirement using the ‘year of retirement’ as the reference category, we fitted generalized estimating equation (GEE) models for repeated measures (with binomial distribution and a log link function). A set of covariates was included in the GEE models: geographical area, sex, age (continuous), marital status (married/registered partnership, divorced/widowed and never married), educational level (low, intermediate and high), occupation (ISCO major categories) as baseline covariates and the presence of at least one chronic disease (yes vs. no) as a time-varying covariate.

We conducted stratified analyses according to strata of geographic area, sex, educational level (low, intermediate and high), age at retirement (equal or less and higher than the country-specific median age of retirement), marital status (married/registered partnership and divorced/widowed) and occupation type (non-manual workers and manual workers) to verify the presence of potential effect modification or confounding factors on the risk of depression and suicidality. We assessed the heterogeneity across strata using likelihood ratio tests.

As sensitivity analyses, we carried out the overall analysis among individuals (*n* = 7,400) from the not-vulnerable subgroup and among individuals (*n* = 8,262) who declared not to receive a disability pension.

## Results

**Figure 1.**
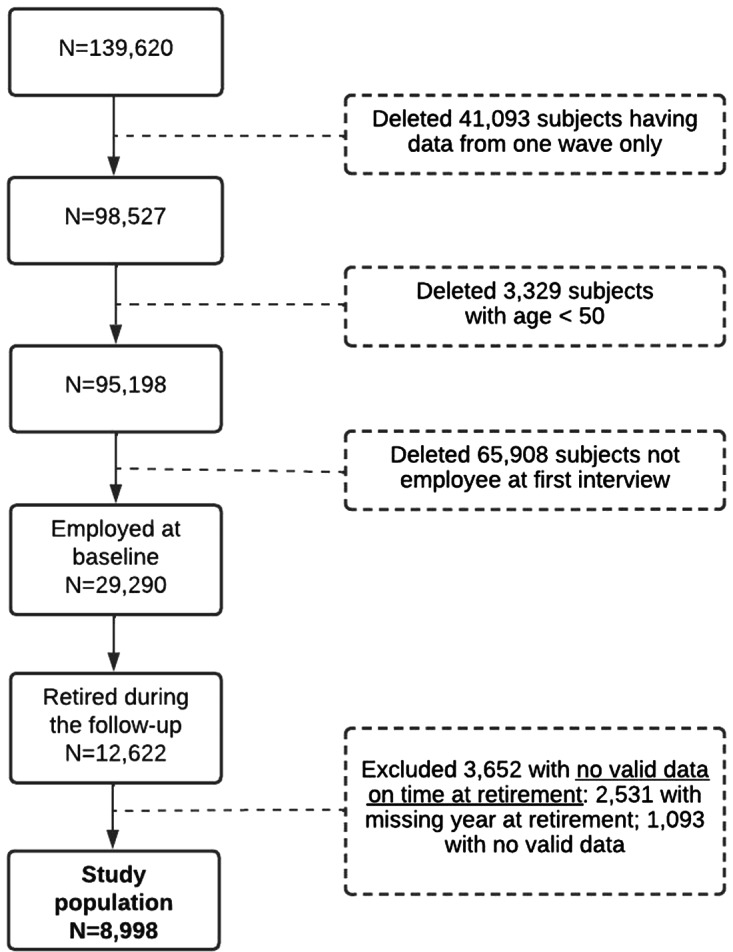
Flowchart of the study cohort selection.

The study cohort selection, including detailed inclusion and exclusion criteria, is reported in Figure 1. From a total of 139,620 subjects included in at least one SHARE wave, we selected a cohort of 8,998 subjects aged 50 and older who declared being ‘employed’ at baseline (i.e., at the first interview) and retired during follow-up. The median age at retirement was 62 years; the maximum follow-up time was 16 years and the median was 9 years.

The distribution of the study cohort according to the main baseline characteristics is reported in Table 1. The study’s cohort consisted of about 53% males, 42.6% individuals aged between 55 and 59 years old (mean age 57 years; standard deviation 4.2), 56.7% and 31% with an intermediate and high education level, respectively, and 83.9% married. Professionals were the most frequent occupation group (15.5%), followed by technicians and associate professionals, clerical support workers, services and sales workers, with proportions of about 13.5%, and managers (10.5%).

**Table 1.** Distribution of the overall study population aged 50 or more (*n* = 8998) according to European geographical area and selected baseline characteristics, 2004–2017.

	*N*	%
*European area*		
North	1626	18.07
West	3738	41.54
South	1480	16.45
East	1880	20.89
Israel	274	3.05
*Sex*		
Male	4797	53.31
Female	4201	46.69
*Age group*		
50−54	2504	27.83
55−59	3831	42.58
≥60 (max: 83)	2663	29.60
*Education level (ISCED)*		
Low (0−1)	1044	11.60
Intermediate (2−4)	5102	56.70
High (5−6)	2787	30.97
Missing	65	0.72
*Marital status*		
Married/registered partnership	7552	83.93
Divorced/widowed	1229	13.66
Never married	107	1.19
Missing	110	1.22
*Occupation (ISCO categories)*		
Managers	944	10.49
Professionals	1397	15.53
Technicians and Associate Professionals	1230	13.67
Clerical Support Workers	1209	13.44
Services and Sales Workers	1223	13.59
Skilled Agricultural, Forestry and Fishery Workers	341	3.79
Craft and Related Trades Workers	872	9.69
Plant and Machine Operators and Assemblers	483	5.37
Elementary Occupations	715	7.95
Armed Forces	88	0.98
Missing	496	5.51

### Risk of depression

**Figure 2.**
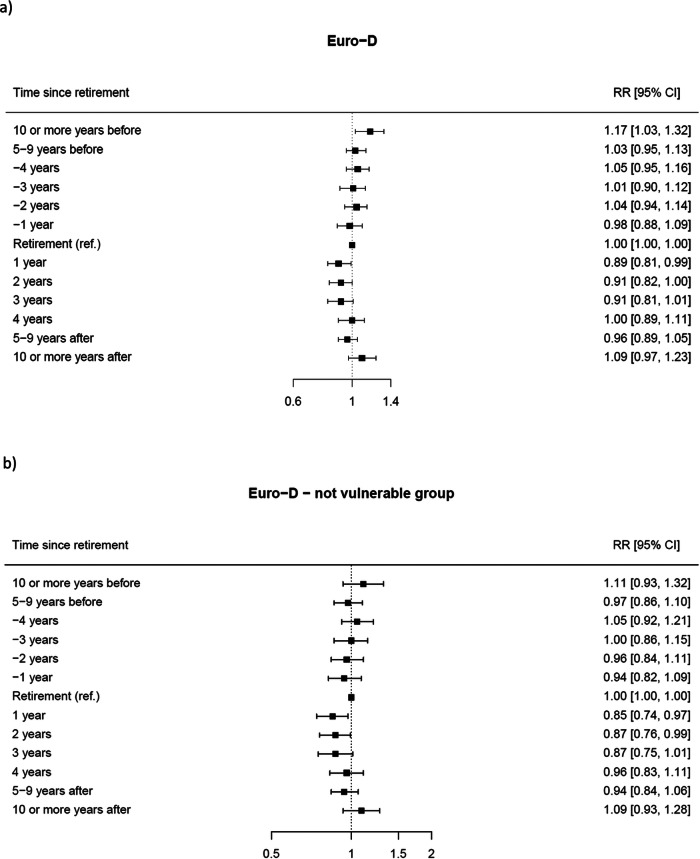
Forest plot of the RR* and corresponding 95% CIs for depression status (Euro-D ≥ 4 vs. Euro-D < 4) at different times before and after retirement (reference category: the year of retirement): (a) overall and (b) not-vulnerable group.

Figure 2 shows the forest plot of the RR and corresponding 95% CI for EURO-D score ≥4 vs. <4 in the whole cohort and in the vulnerable group, at seven time periods before and after retirement. Compared to the year of retirement, the risk of depression resulted 17% higher in the period from 10 years or more before retirement (RR 1.17; 95% CI 1.03–1.32). Conversely, the risk started to decline after retirement, by 11% after 1 year (RR 0.89; 95% CI 0.81–0.99), by 9% both after 2 years (RR 0.91; 95% CI 0.82–1.00) and after 3 years (RR 0.91; 95% CI 0.81–1.01). Reductions in the risk of depression in the immediate years following retirement remained when considering only the not-vulnerable subgroup as a sensitivity analysis. The risk decreased by 15% after 1 year (RR 0.85; 95% CI 0.74–0.97) and 13% after 2 years (RR 0.87; 95% CI 0.76–0.99) from retirement.

**Figure 3.**
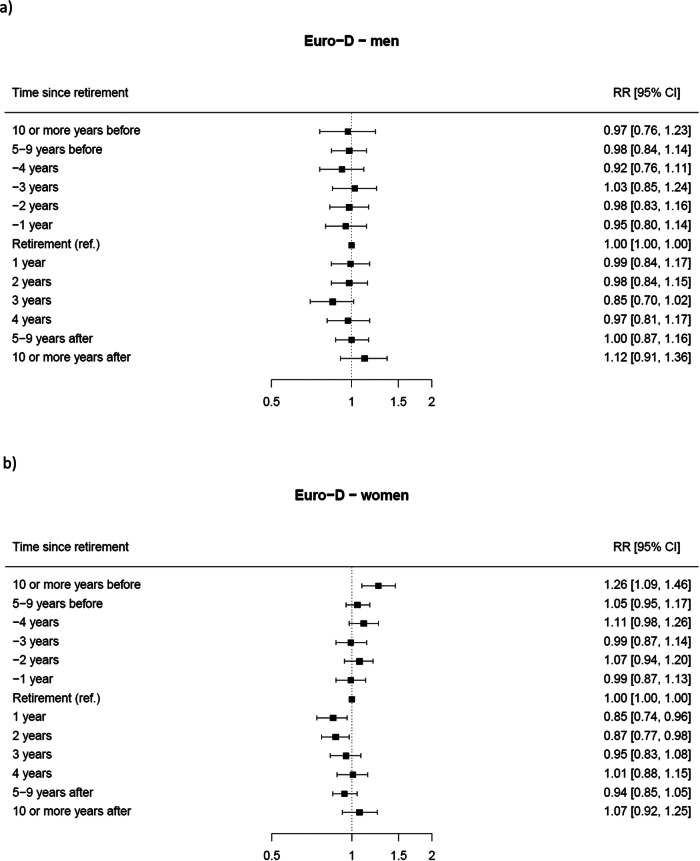
Forest plot of the stratified RR* and corresponding 95% CIs for depression status (Euro-D ≥ 4 vs. Euro-D < 4) at different times before and after retirement (reference category: the year of retirement) by sex: (a) men and (b) women.

**Figure 4.**
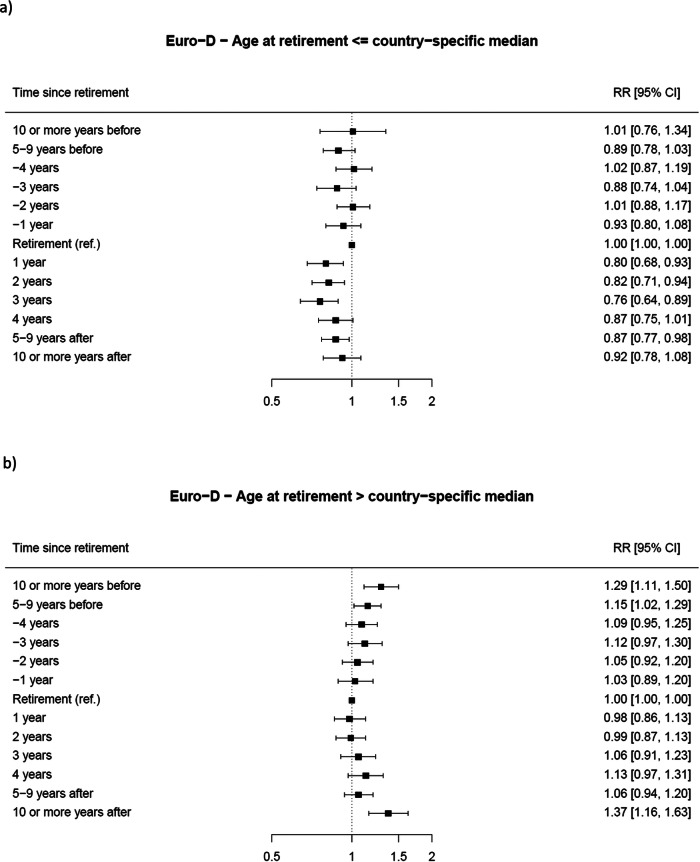
Forest plot of the stratified RR* and corresponding 95% CIs for depression status (Euro-D ≥ 4 vs. Euro-D < 4) at different times before and after retirement (reference category: the year of retirement) by age at retirement: (a) age equal or less than the country-specific median and (b) age greater than the country-specific median.

Results from the stratified analyses are reported in Figure 3 by sex and Figure 4 by age at retirement.

*Estimates were obtained from a GEE model for repeated measures, adjusted by geographical area, sex, age group (50–54, 55–59 and 60+), marital status (married/registered partnership, divorced/widowed and never married), educational level (low, intermediate and high), occupation (ISCO major categories) as baseline covariates and the presence of at least one chronic disease as time-varying covariate (yes vs. no).

As for sex, the associations remained consistent among females for whom the risk of depression was 26% higher 10 years or more before retirement (RR 1.26; 95% CI 1.09–1.46), while it decreased by 15% 1 year after retirement (RR 0.85; 95% CI 0.74–0.96) and by 13% after 2 years (RR 0.87; 95% CI 1.09–1.46).

Decreased risk of depression at 1, 2 and 3 years after retirement was also found among individuals whose age was below (or equal to) their country-specific median retirement age, with RRs of 0.80 (95% CI 0.68–0.93), 0.82 (95% CI 0.71–0.94), and 0.76 (95% CI 0.64–0.89), respectively. In contrast, among subjects whose age was over their country-specific median retirement age, the risk of depression showed an increase at 10 years or more before retirement (RR 1.29; 95% CI 1.11–1.50), between 5 and 9 years before retirement (RR 1.15, CI 1.02–1.29) and at 10 or more years after retirement (RR 1.37, CI 1.16–1.63).

Further stratified analyses are reported in the Supplementary Figure S1 by education, Supplementary Figure S2 by marital status and Supplementary Figure S3 by occupation. We reported patterns consistent with those of the whole cohort among strata of individuals with an intermediate level of education, married retirees and manual workers. However, estimates between strata were not statistically heterogeneous.

### Suicidality

**Figure 5.**
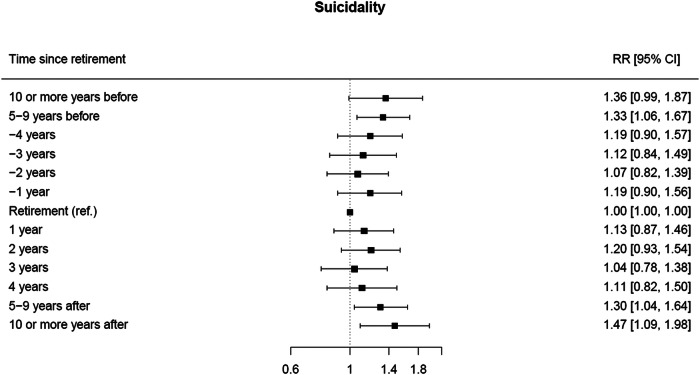
Forest plot of the RR* and corresponding 95% CIs for suicidality risk at different times before and after retirement (reference category: the year of retirement).

As compared to the year of retirement, the risk of suicidality in the whole cohort (Figure 5) was 33% higher between 5 and 9 years before retirement (RR 1.33; 95% CI 1.06–1.67), 30% higher between 5 and 9 after retirement point (RR 1.30; 95% CI 1.04–1.64) and 47% higher at 10 years or more after retirement (RR 1.47; 95% CI 1.09–1.98).

The stratified analyses by sex are reported in Supplementary Figure S4a and S4b. Suicidality risk was 54% higher at 2 years after retirement (RR 1.54; 95% CI: 1.05–2.26) among men. Moreover, an increased risk persisted from the second year onwards, although it was statistically significant only at 10 years or more after retirement (RR 1.78; 95% CI: 1.11–2.86). Among females, the risk of suicidality was 55% higher at 10 years or more before retirement (RR 1.55; 95% CI: 1.07–2.25) and 35% higher between 5 and 9 years before retirement (RR 1.35; 95% CI: 1.01–1.80).

The results of the sensitivity analysis are reported in Supplementary Figure S5. Among the not-vulnerable subgroup, as compared to the year of retirement, the risk of suicidality was 54% higher between 5 and 9 years after retirement (RR 1.54; 95% CI 1.10–2.15) and 92% higher after 10 or more years (RR 1.92; 95% CI 1.24–2.96).

## Discussion

We assessed the short- and long-term effects of retirement transition on depressive symptoms and suicidality in a large European longitudinal cohort of almost 9,000 individuals who transitioned from work to retirement during the study period. Overall, compared to the year of retirement, we observed a 9% to 11% reduction in the risk of depression in the following 3 years. Moreover, in the final 9 years of employment, we observed a risk reduction compared to the earlier period, suggesting a possible anticipatory effect. A sensitivity analysis performed excluding subjects who reported a diagnosis of depression over the study period showed comparable results. When stratified by selected covariates, estimates remained consistent among females, married individuals, those with an intermediate or higher level of education, former manual workers and those who retired at or before their country’s median retirement age. By contrast, an increase in risk was particularly notable among former non-manual workers and late retirees from the tenth year after retirement. As for suicidality, our results showed an evident increase in risk only a few years after retirement: in particular, 30% increased risk 5–9 years after retirement and 47% 10 or more years after.

Our systematic review and meta-analysis published in 2021 suggested that retirement might be followed by a reduction in the risk of depression of around 20% (Odone *et al.*, 2021). However, the results of the present study suggest that the supposed retirement’s protective effect may be limited to a relatively narrow time window. According to a well-established interpretive model of the adjustments to life transitions, retirement, in the short term, can be followed by a ‘honeymoon phase’ in which retirees may experience relief from occupational stress and enjoy a momentary improvement in mood due to new-found freedom and increased availability of leisure time (Atchley, 1976). Indeed, although employment is generally associated with a better mental health status than unemployment (Modini *et al.*, 2016), some work-related factors have been linked to different adverse outcomes, including psychological distress (Harvey *et al.*, 2017). In particular, individuals who perceive their job as more draining and demanding experience increased levels of anxiety and depression (Mc Carthy *et al.*, 2017; Mezuk *et al.*, 2011) and might therefore have a considerable benefit from retirement. Furthermore, due to the disruption of daily routines, consolidated social patterns and lifestyles, retiring can offer the chance to spend time on leisure, social and healthy activities that were sacrificed during working life, fostering an improvement in psychological well-being (Vigezzi *et al.*, 2021). However, it is possible that this initial phase also brings with it certain expectations that, after a while, may become unreasonable and over-optimistic, resulting in disenchantment (Sohier *et al.*, 2021). Other studies confirm that mental health advantages are most evident in the early retirement years (Fleischmann *et al.*, 2020). It is noteworthy that, compared to the previous period, experiencing depressive symptoms in the last years of employment seems to decrease, suggesting that, since the statutory pension age is in most cases predictable, workers may experience anticipatory psychological relief (Vigezzi *et al.*, 2021).

Several factors are likely to influence the reduced risk of depression observed after retirement. In 2007, van Solinge proposed that the relationship between transition to retirement and mental health status not only changes over time but is the result of a complex balance of individual and environmental factors, including demographic aspects, work and retirement transition characteristics and access to cultural and socioeconomic resources (van Solinge, 2007).

First of all, the role of age must be considered: available evidence reports that depression prevalence decreases with ageing (WHO, 2017). Hence, we adjusted our models for age at baseline in order to purge its potentially confounding effect, thus highlighting the independent effect of retirement on mental health status.

Second, according to the ‘role hypothesis’, retirement causes people to lose part of their social role, networks and stimulation, which can lead to anxiety and depressive symptoms and lower levels of well-being. Several authors observed that men are more susceptible to this phenomenon than women (Wang *et al.*, 2011) with a mitigation of the possible beneficial effects of the 'honeymoon phase'. This may be explained by the fact that distinct coping mechanisms facing the transition to retirement may result from the primary roles traditionally played at work and home by men and women, respectively (Forman-Hoffman *et al.*, 2008; Moen, 1996). Our results are in accordance with this theory, showing a statistically significant protective effect of the transition to retirement on depressive symptoms only among women.

Third, although retiring too far in advance of cultural and institutional timetables is thought to have a detrimental effect on health (Calvo *et al.*, 2012), we report that those who withdrew from the labour market before their countries’ median age at retirement experience a 3-year significant ‘honeymoon phase’. Moreover, in contrast with late retirees, these subjects did not show a sustained increase in the risk of depression 10 or more years after pension. In view of the recent pension reforms implemented across Europe, which considerably raised the pensionable age, our findings suggest that greater flexibility in the timing of retirement may positively impact mental well-being, protecting against the risk of depression, especially in the long run. As a matter of fact, flexibility is strictly related to the desirability and degree of control of the transition, considering that involuntary retirement generally has a negative impact on mental health status (Mosca and Barrett, 2016).

Moreover, the greater benefit in terms of mental health outcomes experienced by manual workers after retirement compared to non-manual workers confirms recent evidence from the literature highlighting the crucial role of employment history, time and workload on the risk of mental health disorders’ onset in this phase of life (Ardito *et al.*, 2020; de Wind *et al.*, 2014).

Finally, access to social, cultural and financial resources can also mitigate the impact of the psychological consequences of retiring (Arias-de la Torre *et al.*, 2018; Choi *et al.*, 2013; Deeg and Bath, 2003; Park and Kang, 2016; Sabbath *et al.*, 2015; Shiba *et al.*, 2017). In line with our hypotheses, we found that marriage and intermediate or higher education (i.e., a reliable proxy of socioeconomic status) emerged as possible protective factors from the adverse effects of retirement on mental health.

Although previous research has generally linked retirement to an increased risk of suicide and suicidal behaviours (Harwood *et al.*, 2006; Minayo *et al.*, 2012; Page *et al.*, 2021; Schneider *et al.*, 2011), our results showed a significant increase in the risk of experiencing suicidal thoughts only at least 5 years after retirement, supporting the hypothesis that the first years after the end of working life are accompanied by a reduction in depressive symptoms. Moreover, these findings on the increased suicidal ideation in late life, several years after pension, are in line with the alarming rate of older people committing suicide, particularly those over 80 years of age (Naghavi, 2019). While depression is a major risk factor for suicidal ideation, its role has been generalised too far (De Leo, 2019), especially in old age, when its prevalence tends to decrease (WHO, 2017). Other factors, including social determinants of health (Wilkinson and Marmot, 2003) and the transition to retirement among them, might play a role. By leveraging them, we could improve older people’s mental health (De Leo, 2022). After stratification by sex, a significant risk increase in suicidal ideation was observable among men even 2 years after retirement. This latter finding reaffirms the concept that the positive effects of retirement on mental health may be less pronounced in men, and it should presumably still be read in the context of the ‘role hypothesis’ and related sex differences.

This study needs to be interpreted in light of several strengths and limitations. Among its strengths, we acknowledge the use of high-quality data with standard protocols and definitions across countries and waves, the adoption of a validated evidence-based screening tool for depression risk and the conduction of both stratified and sensitivity analysis to test the role of determinants and our results’ consistency in selected subgroups. More importantly, a longitudinal cohort analysis allowed us to study the most extended possible follow-up period within SHARE. Limitations of our study include possible information bias due to the self-reported symptoms assessment and potential recall bias. Moreover, differences in retirement policies across the included countries that may have affected exits from employment should have been considered. Given that the study cohort was selected following rigorous but subjective criteria, as done in previous studies which analysed the same population (Bertuccio *et al.*, 2023), the representativeness of the cohort and the generalizability of our results might be limited.

If confirmed by future studies, our findings could support evidence-based health and welfare policies as well as the planning and implementation of appropriate and targeted interventions centred on the most vulnerable individuals. On the one hand, primary mental health interventions might fruitfully support social roles and promote healthy lifestyles (Lindwall *et al.*, 2017) so as to reduce the risk of depression and suicide in old age (Lapierre *et al.*, 2011). More multidisciplinary efforts should be devoted to boosting and prolonging in the long term the benefits of retirement on mental health status, exploiting life-course transitions’ tendency to bring along health-related changes and synchronising them with public health, prevention and health promotion interventions (Ben-Shlomo and Kuh, 2002; Heaven *et al.*, 2016). On the other hand, although the pensionable age is set to increase in Organization for Economic Co-operation and Development (OECD) countries (Eisen *et al.*, 2022), our findings suggest that the adverse health effects of late retirement may have been underestimated and should be taken into account in cost-effectiveness assessments of public policies.

## Conclusions

Our data complement the accumulating evidence on the impact of retirement and transition to retirement on mental health. More flexibility in the timing of retirement should be granted according to factors influencing mental health (e.g., sex, education, type of work and marital status) in order to reduce the burden of mental health in old age and reduce the risk of depression and suicidality.

In our ageing society, despite many countries implementing budget cuts to welfare, it is essential to evaluate retirement policies’ impacts on healthcare and social support systems and invest in older adults’ health and well-being, promoting a culture of resilience and adaptation to the different stages of life and the changes that come with advancing age.

## Data Availability

The datasets supporting the conclusions of this study are publicly available from SHARE Research Data Center (https://releases.sharedataportal.eu/) upon request.

## Supplementary material

For supplementary material accompanying this paper visit http://doi.org/10.1017/S2045796023000239. click here to view supplementary material
