# Supplementary material for: Transition to retirement impact on risk of depression and suicidality: results from a longitudinal analysis of the Survey of Health, Ageing and Retirement in Europe (SHARE)
Source: Epidemiol Psychiatr Sci. 2023 May 11;32:e34. doi: 10.1017/S2045796023000239 (PMC10227535; doi:10.1017/S2045796023000239)
Supplement: Supplementary file 1 [file S2045796023000239sup001.docx]

**SUPPLEMENTARY MATERIALS**

**Supplementary Figure 1.** Forest plot of the stratified relative risk (RR)* and corresponding 95% confidence intervals for depression status (Euro-D ≥4 vs Euro-D<4) at different times since retirement (reference category: the year of retirement) by education. Panel a) low; panel b) intermediate; c) high.


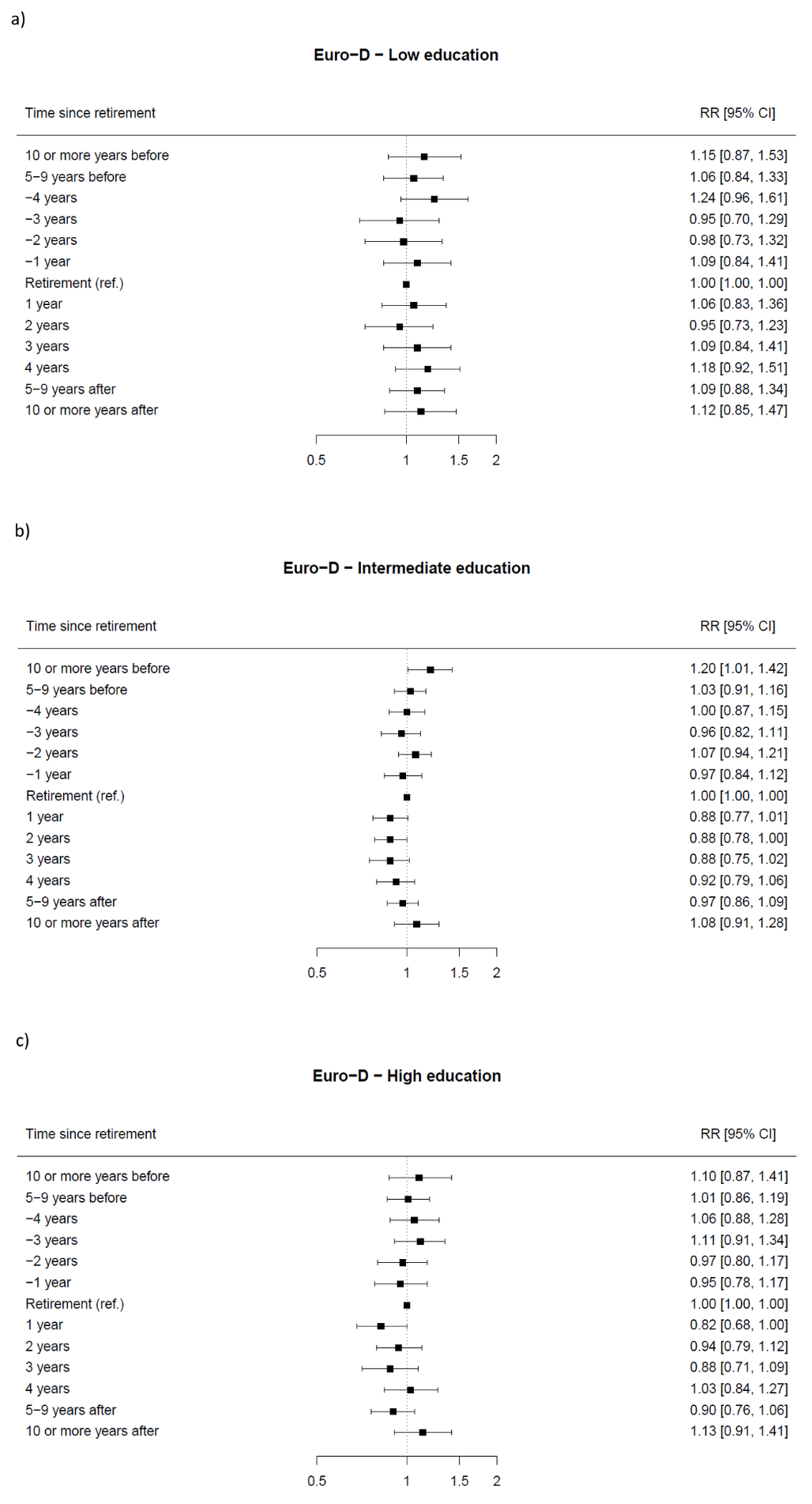


**Supplementary Figure 2.** Forest plot of the stratified relative risk (RR)* and corresponding 95% confidence intervals for depression status (Euro-D ≥4 vs Euro-D<4) at different times since retirement (reference category: the year of retirement) by marital status. Panel a) married/registered partnership; panel b) divorced/widowed.


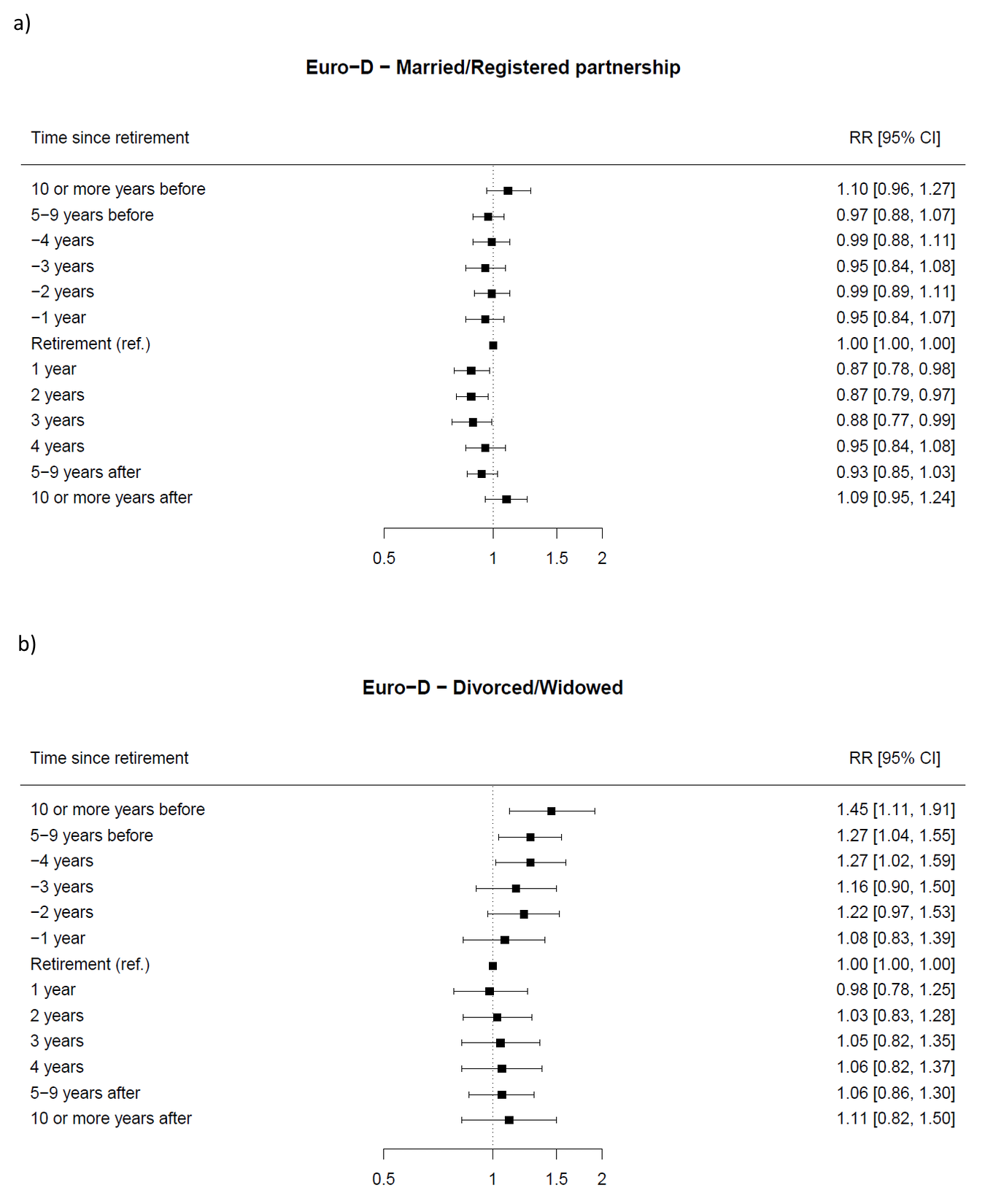


**Supplementary Figure 3.** Forest plot of the stratified relative risk (RR)* and corresponding 95% confidence intervals for depression status (Euro-D ≥4 vs Euro-D<4) at different times since retirement (reference category: the year of retirement) by occupation type. Panel a) non-manual workers; panel b) manual workers.


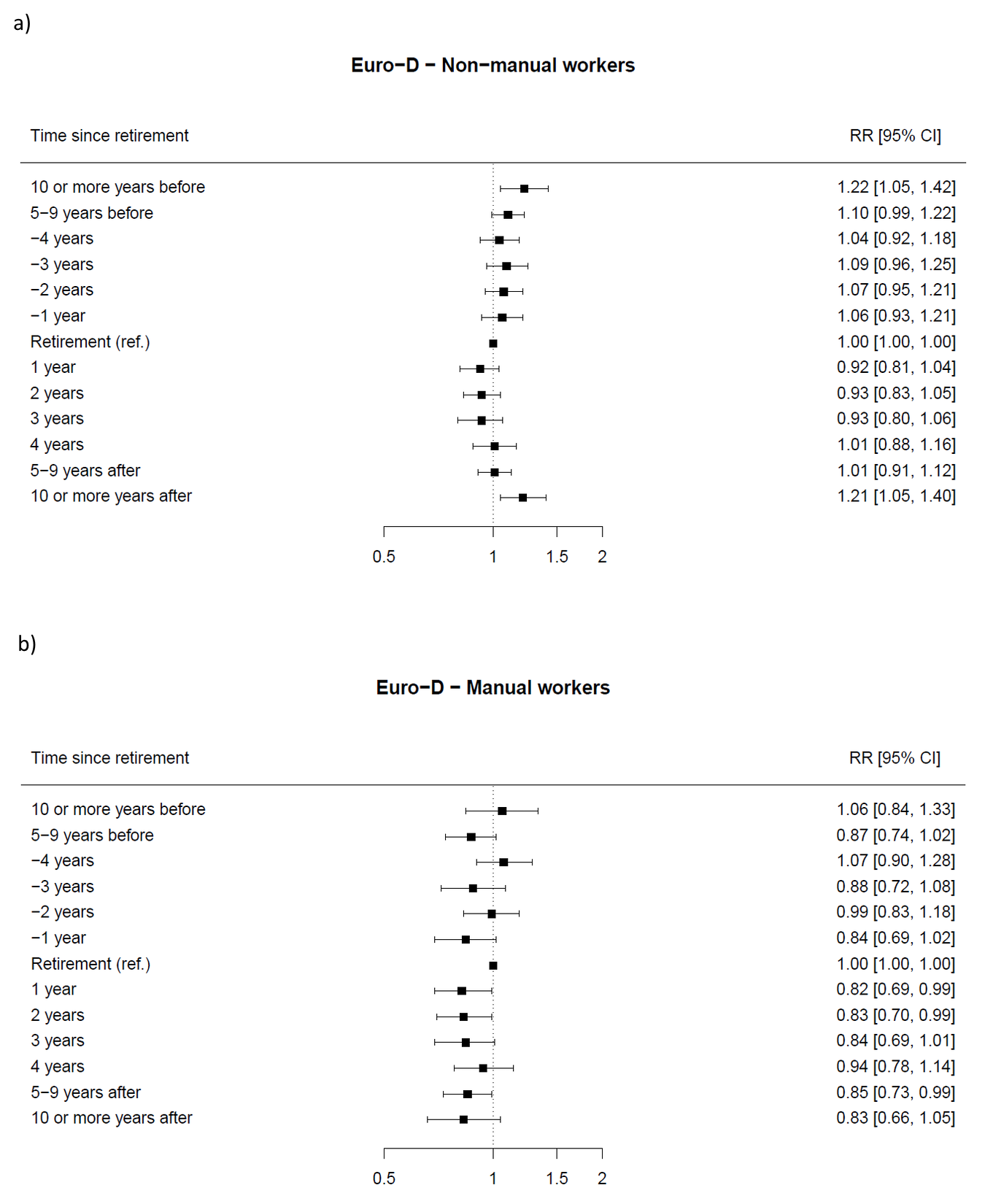


**Supplementary Figure 4.** Forest plot of the stratified relative risk (RR)* and corresponding 95% confidence intervals for suicidality at different times since retirement (reference category: the year of retirement) by sex. Panel a) men; panel b) women.

**
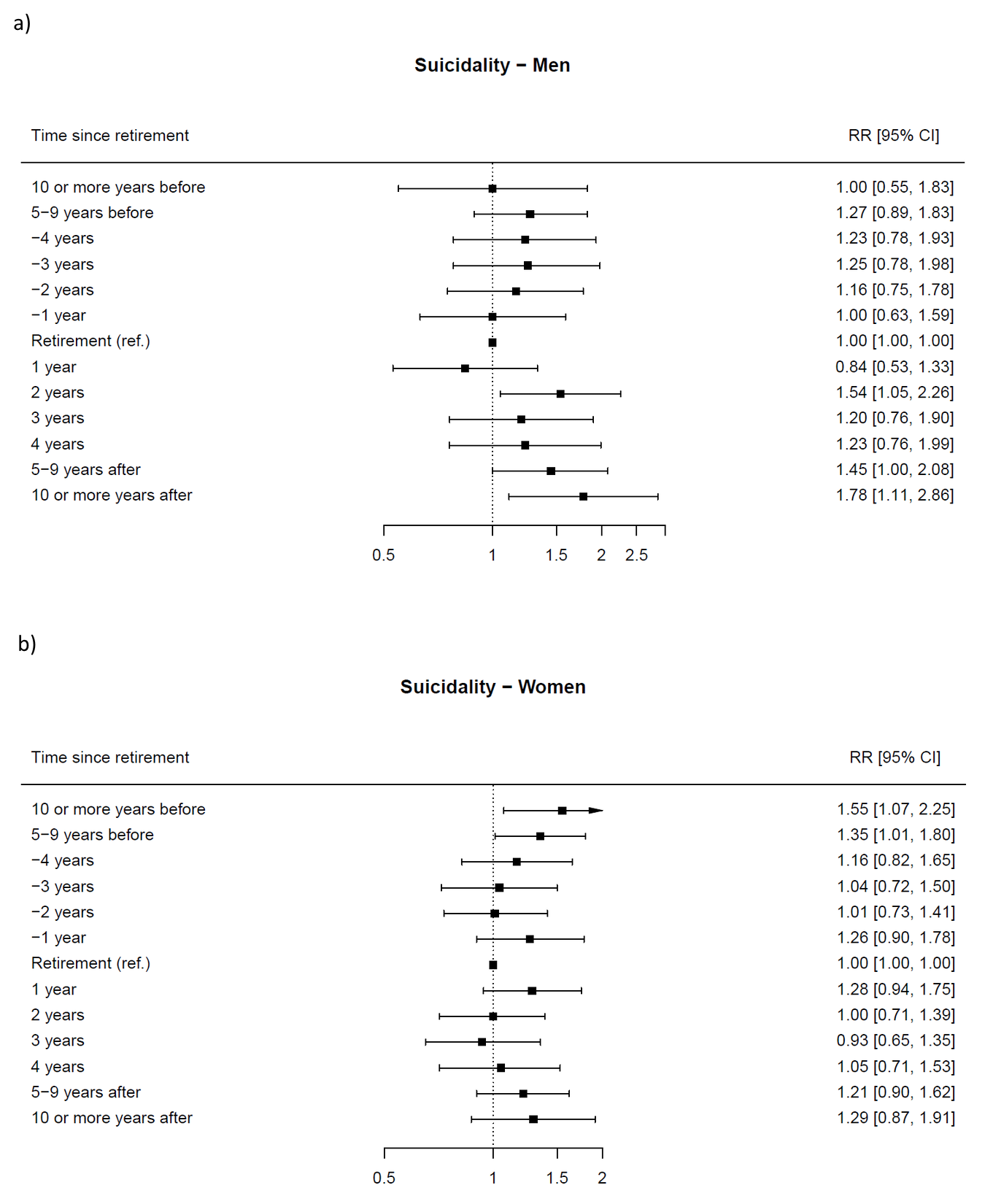
**

**Supplementary Figure 5.** Forest plot of the stratified relative risk (RR)* and corresponding 95% confidence intervals for suicidality risk at different times since retirement (reference category: the year of retirement) in the not-vulnerable group.

**
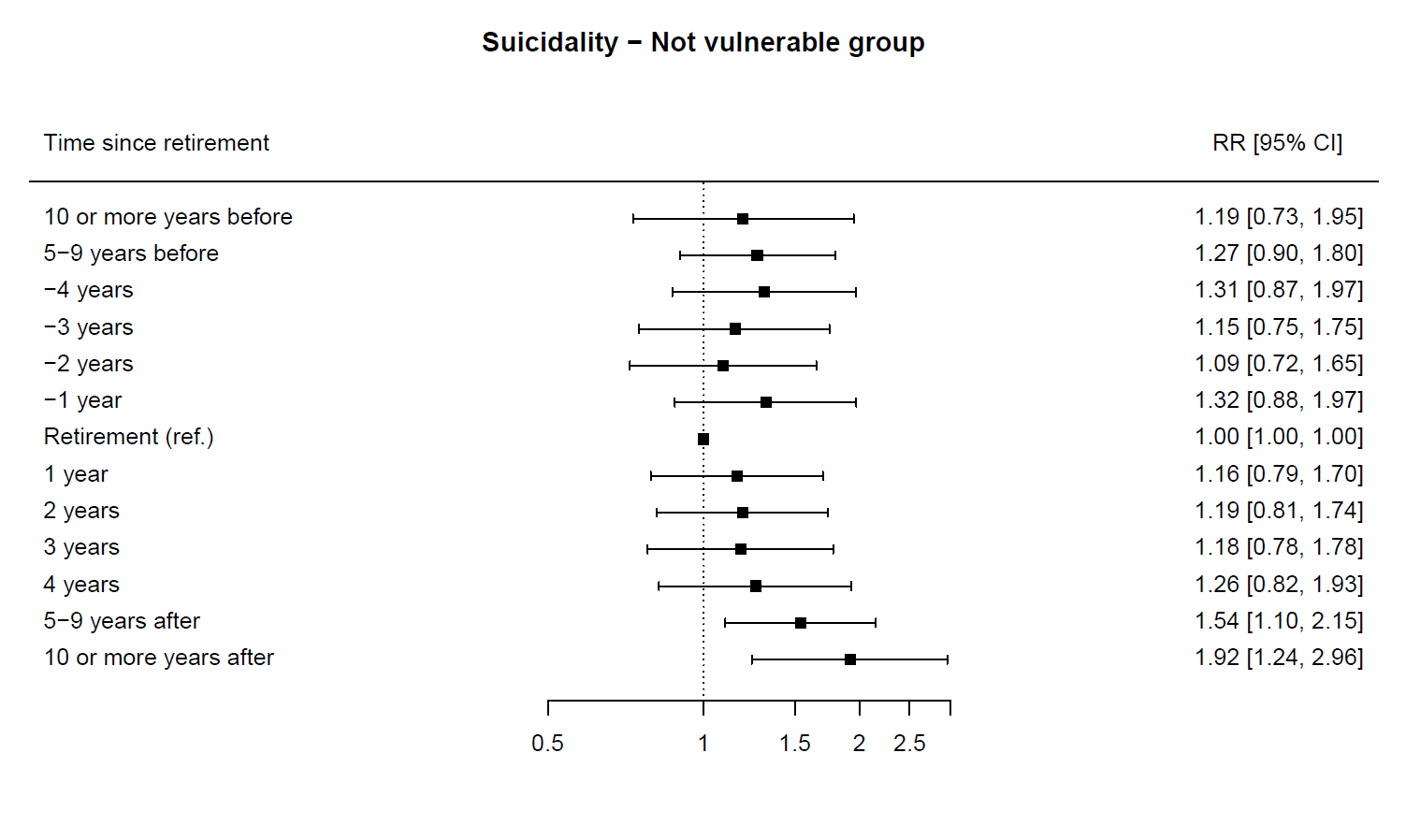
**
